# Spontaneous Miscarriage Management Experience: A Systematic Review

**DOI:** 10.7759/cureus.24269

**Published:** 2022-04-19

**Authors:** Angela L Ho, Algeny Hernandez, John M Robb, Stephanie Zeszutek, Sandy Luong, Emiru Okada, Karan Kumar

**Affiliations:** 1 Obstetrics and Gynecology, Touro College of Osteopathic Medicine, Middletown, USA; 2 Obstetrics and Gynecology, University of California Irvine, Irvine, USA; 3 Obstetrics and Gynecology, Drake University, Des Moines, USA; 4 Surgery, Touro College of Osteopathic Medicine, Middletown, USA

**Keywords:** postpartum mental health, postnatal depression, women's mental health, spontaneous abortion, miscarriage

## Abstract

Background: The estimated frequency of spontaneous miscarriage is about a quarter of all clinically identified pregnancies in the United States. Women typically go to the emergency department (ED) or outpatient clinic when they experience symptoms, including but not limited to vaginal bleeding, abdominal pain, and contractions. The care that is provided varies from place to place.

Methods: Researchers searched articles from 2010 to 2021 for reports mentioning treatment for spontaneous abortion. Search terms included "miscarriage aftercare" and "spontaneous abortion care," seeking articles addressing the psychological effects of miscarriage and reporting patient experiences in different clinical settings. Data were independently reviewed, graded for evidence quality, and assessed for risk bias using the AMSTAR checklist.

Results: The search strategy yielded 2,275 articles, six of which met the inclusion criteria. Conservative, medical, and surgical management were provided, with surgical management being more common among women with higher education and socioeconomic status. All qualitative studies reported dissatisfaction with care provided in the emergency department, partially due to a lack of emotional support. Structured bereavement intervention was beneficial for women experiencing early pregnancy loss and led to fewer reports of despair. The quantitative studies referenced interventions that aided patients in coping with pregnancy loss and identified several factors influencing the type of treatment received as well as the patient's ability to cope with feeling depressed following a miscarriage.

Conclusion: Psychological management is not regularly addressed in the emergency department, and protocols including bereavement education for healthcare providers as well as patient involvement in management would improve the overall patient experience with spontaneous miscarriage care.

## Introduction and background

Spontaneous miscarriage is the loss of a pregnancy prior to 20 weeks of gestation, which is the most common complication of pregnancy [1]. Spontaneous miscarriages occur in about 20% of pregnancies in the United States [2]. Women experiencing miscarriage may be treated in a multitude of places: prenatal clinics, the emergency department (ED), gynecologic outpatient offices, same-day surgical departments, or the labor and birth unit [3]. Nearly, 40% of women, going to the ED to manage their miscarriage is primarily for convenience or active vaginal bleeding [4]. Although family practice medical offices can offer comprehensive spontaneous abortion management or treatment, few do [5]. Oftentimes, women who experience spontaneous miscarriage will have long-lasting grief and psychological sequalae [4]. As many as 50% of miscarrying women suffer psychological morbidity months after loss and symptoms could persist up to 1 year after miscarriage [6]. Experiencing a miscarriage can lead to mental health complications such as moderate to severe anxiety, moderate to severe depression, and post-traumatic stress disorder [7,8]. There are reported feelings of shame, fear, guilt, helplessness, and grief following a miscarriage [4]. Women of lower socioeconomic status, those with a history of psychiatric illness, and/or those lacking social support are more likely to experience severe psychological distress post-miscarriage [9]. 

Bergner et al. found that maladaptive coping strategies increased the risk for depression at seven months postmiscarriage, and that carried over into women’s subsequent new pregnancies [10]. Research shows that some women mourn and cope with their miscarriage alone because of societal/cultural ramifications of pregnancy loss, which impose additional distress on already vulnerable women [11]. Further evidence suggests that women who are insufficiently supported by their partner, family, or social network are more likely to develop severe grief reactions and psychopathology than those with supportive relationships [12-14].

Experiences during health care encounters can also add stressors to a traumatic experience. Patients who experienced a lack of emotional support expressed feelings of being alone and unheard while they were in a confused and fearful state [4]. Psychological and supportive care following a miscarriage has not been extensively studied and lacks single-blinded randomized controlled trials in this area of research [15]. Interventional studies are also sparse.

The purpose of this review is to identify, evaluate, and summarize the findings of all relevant individual studies regarding spontaneous miscarriage psychological treatment and patient experiences in various clinical settings in the USA.

## Review

Inclusion criteria

This study was conducted as a systematic review utilizing PubMed and EBSCO databases. The search terms were "miscarriage care" and "spontaneous abortion care." These keywords were used to search for articles published after 2010 whose full text was available in English. The search was restricted to human research articles, including case reports, clinical trials, and comparative studies published in peer-reviewed journals. Only studies including female participants between the ages of 18 and 64 who experienced spontaneous miscarriage were included. We included studies that specified miscarriage aftercare provided in an inpatient (emergency department) and outpatient setting (obstetric/gynecology clinic), studies highlighting different medical treatments for miscarriage, studies reporting patient experiences in different clinical settings, and studies conducted in the US.

Exclusion criteria

Studies that did not specify any clinical diagnosis of miscarriage, non-peer-reviewed literature, systematic reviews, opinion articles, and editorials were excluded.

**Figure 1:**
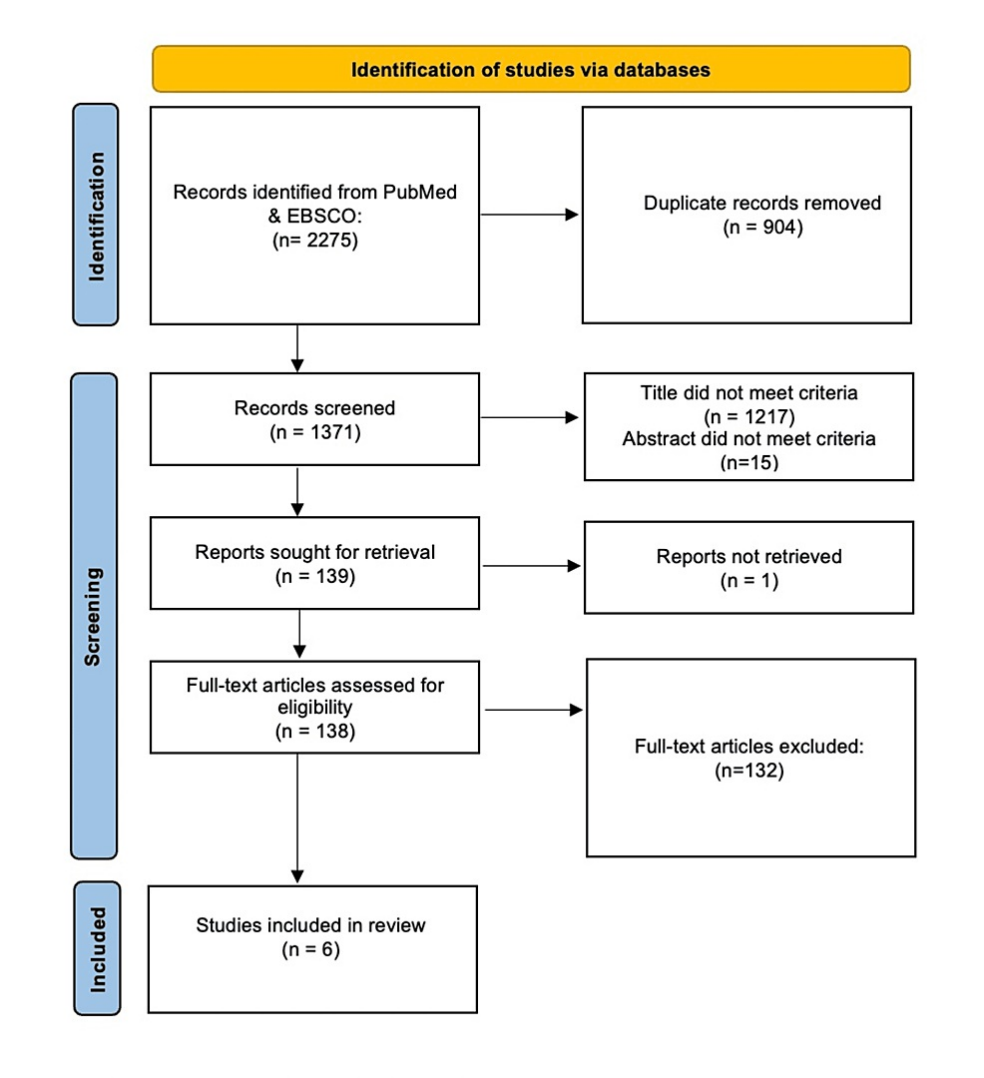
PRISMA flow chart of literature search PRISMA: Preferred Reporting Items for Systematic Reviews and Meta-analyses

The search took place in September 2021. The selection of studies for this systematic review was presented using the Preferred Reporting Items for Systematic Reviews and Meta-analyses guidelines (Figure 1) [16]. Data including samples, study design, clinical setting, primary results, and main findings were extracted from eligible studies (Table 1). The quality of our systematic review was assessed by the AMSTAR checklist [17].

**Table 1:** Characteristics of studies reporting spontaneous miscarriage treatment in U.S.

Author	Publication year	Location	Study design	Sample size
Johnson and Langford [18]	2015	ED	Prospective	40
Miller et al. [7]	2019	ED or ambulatory clinics	Mixed methods	54
Punches et al. [19]	2019	ED	Qualitative	8
Schreiber et al. [20]	2016	ED	Mixed methods	55
Baird et al. [4]	2018	ED	Qualitative	67
Wilson et al. [21]	2016	Pregnancy loss center	Prospective	70

Of the 2,275 articles identified, 904 were removed as duplicates, and 1371 were screened. Of these, 1217 were excluded based on title review, and an additional 15 were excluded during the subsequent abstract review. The remaining 139 reports were sought for retrieval, with one report unable to be acquired. Full-text assessment of the remaining 138 accessed articles yielded 132 that failed to meet eligibility criteria, with many of the studies taking place outside of the US. Ultimately, six articles were selected. Of the six included studies, four contained quantitative analyses (Table 2), and four contained qualitative analyses (Table 3). Two of the six studies included in this review contained both qualitative and quantitative data.

**Table 2:** Summary of quantitative studies investigating spontaneous miscarriage treatments

Author	Treatment	Main outcome measures	Primary results
Johnson and Langford [18]	Secondary bereavement intervention	Perinatal grief scale	Women who received bereavement intervention immediately after miscarriage were able to better cope with pregnancy loss.
Miller et al. [7]	Emergency department or ambulatory-only care	Time to miscarriage resolution; number of health care system interactions; and number of care teams	Patients seeking miscarriage care in the ED were likely lower socioeconomic class and psychosocially vulnerable. The median time to miscarriage resolution was 11 days for women treated in the ED and 8 days who were treated in an ambulatory setting. Patients treated in the ED were more likely to be younger (28.3 vs. 34.0), of black race, uninsured or insured through Medicaid, and more likely to meet criteria for post-traumatic stress disorder vs. patients treated in ambulatory clinics.
Schreiber et al. [20]	Expectant, medical, surgical	Maternal demographics	Surgical management was more likely in women with higher education, higher monthly income and less likely to report depression. Multigravidas were more likely to stick with their initial treatment choice after counseling than primigravida.
Wilson et al. [21]	Doula support	28-item brief cope score, 28-item empowerment score, 10-item assessment of emotional state	Doula support does not significantly affect physical discomfort during surgical management of spontaneous abortion. There are no statistically significant differences in satisfaction, emotional response, sense of empowerment or ability to cope between women who received doula support and women who received routine care. Doula support during office uterine aspiration is desired during office uterine aspiration for early pregnancy loss.

**Table 3:** Summary of qualitative studies investigating spontaneous miscarriage treatments

Author	Study Goal	Primary Results
Punches et al. [19]	To understand the perspectives of women who undergo pregnancy loss treatment in the ED on provision of care	Participants reported frustration with the environment of the ED, including lack of privacy and provider unawareness of miscarriage patient needs. Poor communication was described between providers and patients, especially delayed communication of diagnosis.
Baird et al. [4]	To understand why women present to emergency department for spontaneous abortion care, how patients perceive counseling taken place there, and overall experience during and after visit	Many participants reported chaos, lack of information or lack of emotional support. Abnormal vaginal bleeding was the driving factor for seeking care in the ED. Many women reported feeling unsure of next steps and what to expect following the ED visit.
Schreiber et al. [20]	To assess what drives satisfaction with spontaneous miscarriage care	Participants were frustrated with the time of obtaining definitive diagnosis. Prior pregnancy experiences affected the patients’ miscarriage management decision.
Miller et al. [7]	To detail the experiences of patients presenting with miscarriage in ED or ambulatory clinic settings	Participants were more satisfied in ambulatory care settings, citing perceived efficiency of care and confidence in diagnosis. Patients in ED settings were dissatisfied with the lengthy timing of diagnosis communication and inadequate compassion received from care providers.

Spontaneous miscarriage treatment provided

The included studies evaluated various aspects of miscarriage treatment and management. Two specifically assessed outcomes from medical, surgical, and expectant/conservative management. Medical treatment included the use of prostaglandin analogs, such as misoprostol. Schreiber et al. and Wilson et al. utilized dilation and curettage and/or dilation and evacuation [20,21]. 

The selection of expectant, medical, or surgical treatment for spontaneous miscarriage was influenced by factors such as socioeconomic status, level of education, and prior pregnancies. Patients with a higher level of education and income chose more invasive treatments over medical and expectant care for various reasons, including the need to return to work [20]. Participants of lower socioeconomic status made up most of the population treated in the ED [7,18,22]. Miller et al. found women who are socioeconomically and psychosocially vulnerable are more likely to meet the criteria for post-traumatic stress syndrome (PTSD) three months after miscarriage [7]. Given that miscarriage is a traumatic experience, the development of PTSD post-miscarriage is not surprising.

Although spontaneous miscarriage is a common occurrence, there are few studies conducted in the US that focus on the psychological treatment for spontaneous miscarriage. The review identified two interventions for addressing the psychological effects of miscarriage, including doula assistance or bereavement intervention [18,21]. A doula is a trained layperson who provides continuous support to a woman throughout her pregnancy, including praise, encouragement, comfort measures, explanations about the labor progress, and other information pertaining to pregnancy and delivery [23]. Johnson’s study included a one-hour bereavement intervention based on Guidelines for Medical Professionals Providing Care to the Family Experiencing Perinatal Loss, Neonatal Death, SIDS, or other Infant Death, which was provided for the treatment group. The bereavement protocol included: (a) acknowledgment of pregnancy loss by labeling the patient's room and chart; (b) chaplain services; (c) addressing special requests, including baptism or prayer; (d) a packet of flower seeds of remembrance; (e) a soft plush bear; (f) other physical momentos, if applicable; (g) naming ceremony participation; and (h) a self-addressed sympathy card [18]. A one-week follow-up telephone call upon discharge was also completed to reinforce information from the bereavement intervention and encourage women to seek continued emotional support [18].

Nevertheless, since women are at risk for various psychopathologies postmiscarriage, a number of generic psychological and psychiatric assessments (i.e., General Health Questionnaire, Hospital Anxiety and Depression scales, coupled with questions about symptoms of trauma, or Perinatal Grief Scale) could provide insight into the psychological needs of these patients [24-26].

Women’s experience with pregnancy loss****

Common themes across the qualitative studies included frustration with the chaotic clinical environment, poor communication, and delays in delivery of diagnosis. Miller et al. and Punches et al. reported mixed experiences in the ED, with some participants satisfied with the providers’ ability to tailor care by allowing time to cope, while others were upset with the multiple staff handoffs, extensive wait times, and lack of privacy in their care [7,19]. Other studies reported unfriendly environments and a lack of emotional support offered by ED staff [4,27]. In the ED, care is often rushed and women are not given the option to see their lost child, leading to emotional turmoil [28]. Sometimes women were told to subside their concerns and felt neglected by healthcare providers who demonstrated a lack of compassion in care [19,29-32]. Many interviewees were confused about the cause of their pregnancy loss and desired a better understanding of the diagnosis and how to prevent future occurrences [4]. Time constraints of ED providers needing to see multiple patients quickly in order to maintain the "flow" of the department are likely a contributing factor in the negative perception [33]. 

In contrast, patients who received miscarriage treatment in an ambulatory clinic had a clearer and more streamlined experience with their diagnosis and treatment options. [7]. Overall patient satisfaction with care is associated with the presence of supportive staff and with the dissemination of sufficient information regarding miscarriage [20].

Benefits of psychological intervention and emotional support

It can be a traumatizing experience to have to go to a healthcare facility to address a miscarriage. Miscarriage is known to have negative psychological effects on women, including clinical symptoms of depression and anxiety that may occur within the first-week post-miscarriage, and the emotional experience can persist even after the grief subsides [34,35]. Active grieving is expected following a spontaneous miscarriage, and when coping strategies fail, feelings of despair typically set in [36]. The DSM-IV includes grief within the description of major depressive episodes combined with weight loss, guilt, insomnia, and thoughts of self-harm/suicide [37]. A study identified a significantly higher annual suicide rate in women who had miscarried within one year prior to their suicide compared to women who had delivered a baby (18.1 out of 100,000 vs. 5.9 out of 100,000); however, this reaction to pregnancy loss is extreme [38]. Research has shown that persistent depression is linked to childless women, which could be a target intervention group for future studies [9].

Johnson and Langford provided evidence that bereavement intervention can help women better cope with early pregnancy loss [18]. Protective factors against post-miscarriage psychiatric illnesses include multiparity, partner/social support, higher level of education and socioeconomic status, and no prior history of mental illness [9,39]. Therefore, women who lack these protective factors will benefit more from bereavement intervention. Women who received the bereavement intervention reported 50% lower levels of despair in comparison to those who did not receive the intervention [18]. Thus, counseling interventions should be offered to all patients due to the prevalence of persistent depression linked with miscarriage [9]. If these services are provided shortly after the miscarriage, they may be effective in lowering grief, depression, and anxiety [40-45].

Wilson et al. found that doula support during surgical management of spontaneous miscarriage had no significant effect on procedural pain score or pre/post-procedure anxiety [21]. Although doula support did not substantially affect women’s emotional responses or ability to cope with pregnancy loss, over 50% of women reported that doulas helped distract them from their negative emotions during the procedure [21]. Many would want a doula in the future, would recommend doula support to a friend having a similar experience, and thought doula services improved their overall experience [21]. Specialized attention from providers can be therapeutic for women experiencing pregnancy loss [46,47]. Thus, doula services have the potential to address women’s unmet emotional needs before, during, and after surgical intervention [48]. Health insurance coverage can be a barrier to obtaining doula services for some women [49,50]. However, doula services have been shown to lower the cost of care for institutions as these services can address emotional and informational support gaps and aid in reducing complications during pregnancy by reducing preterm births, morbidity, and cesarean deliveries [49,50].

Recommendations to improve patient experience

Spontaneous abortion management experience is heavily affected by the efficiency of care, confidence in the quality of care, sensitive health care providers, and effective two-way communication. Emotional support and patient inclusion in the decision-making process are crucial components of patient-centered care. Updates throughout the evaluation process can aid in avoiding misunderstandings and feelings of isolation, which were frequently reported [47]. As a result of a major gap in psychological care in miscarriage management, support services have emerged as an adjunctive social movement [51,52]. Potential interventions to improve patient perception and insight into their own miscarriage care can be implemented by institutions to promote practices that will improve women’s experience when receiving care in the ED. Some ED providers feel unprepared to provide bereavement support; therefore, bereavement education can be administered through structured computer-based education modules periodically throughout training and practice to better care for this population [53-55].

The quality of miscarriage care is significantly better when providers give medical and emotional validation while keeping the patient involved in the clinical course and decision-making [20,56-59]. Patients should be educated and given supplemental education materials routinely during pregnancy on the frequency of miscarriage, potential causes, potential preventative measures, and physical/psychological aftercare - all of which can normalize and destigmatize this natural process [57]. Patients should be informed to first contact their primary care provider or OB/GYN if they develop concerning symptoms in order to assess if emergent interventions are necessary. Information can be provided electronically or physically upon discharge from the ED, including support groups, bereavement services, chaplain services, or any other institutional or area-specific resources. Some evidence supports the effectiveness of these resources for women who experienced neonatal death [60,61]. Communication between the ED and the patients’ primary care or obstetrics provider should be attempted in an effort to aid in continuity of care, subsequent follow-up visits, and telemedicine services.

Limitations to the review

One limitation of this review is the geographical restriction to the US. Only studies published in peer-reviewed journals were included to ensure reporting quality; thus, relevant gray literature was not evaluated for this study.

## Conclusions

Despite the limited number of studies available for this review, the studies that were available highlight important advances that can be made in miscarriage aftercare and where research is lacking. From what we have reviewed, protocols are beneficial for this patient population in settings such as the ED, where medical and psychological needs can be more effectively addressed. It is evident that psychological interventions are beneficial for this patient population. Transparency and patient education regarding what will occur during and after immediate care for spontaneous abortion are beneficial to the patient's mental and physical well-being as well as the overall patient experience.
